# Overseeing health care facilities in Shanghai, China: regulatory regime, activities and challenges of the governmental regulatory system

**DOI:** 10.1186/s12939-019-0979-1

**Published:** 2019-05-24

**Authors:** Yizhong Zhou, Zechang Lu, Zhifeng Yang, Huidi Li, Yingyao Chen

**Affiliations:** 1 Department of Hospital Management, School of Public Health, Key Lab of Health Technology Assessment, National Health Commission, Fudan University, Shanghai, 200032 People’s Republic of China; 2 Department of Medical Service Supervision, Agency for Inspection and Supervision, Shanghai Municipal Health Commission, Shanghai, 200031 People’s Republic of China; 3 Division of Medical Service Supervision, Agency for Inspection and Supervision, Shanghai Yangpu District Health Commission, Shanghai, 200090 People’s Republic of China

**Keywords:** Government regulation, Health care facility, Regulatory activity, Inspection, Administrative penalty, Shanghai

## Abstract

**Background:**

Government regulation has played a crucial role in ensuring the quality, safety and equity of health care. However, few empirical studies have investigated Chinese governmental oversight of health care facilities in terms of regulatory arrangements and approaches. This study aims to explore the regulatory regime and main activities within the health sector in Shanghai, a city featuring abundant health care resources and a complex medical system, to provide policy implications for better regulation and offer valuable reference for elsewhere in China and other developing countries.

**Methods:**

We explored the structure and main activities of government regulation over health care facilities in Shanghai, compared it with the regulatory system in Hong Kong and Taipei through a literature review and analyzed the data on regulatory activities conducted by the local Health Supervision Agencies using descriptive statistical analysis. The data were collected from the Shanghai Statistical Yearbook 2014–2018 and the centralized data bank of the Shanghai Health Supervision Authority.

**Results:**

Shanghai has established a unique governmental regulatory system compared to Hong Kong and Taipei. We found health care facilities in Shanghai underwent less frequent inspections between 2013 and 2017, as average annual inspections at individual facilities decreased from 3.8 to 2.7. The number of annual administrative penalties and notifications issued for accumulating points on local health care facilities’ violations decreased by 24.8 and 40.7%, respectively, and complaints against health care facilities decreased by 29.1% during the study period.

**Conclusions:**

The local governmental regulatory system played a vital role in overseeing the health care facilities and ensuring their legal compliance by exerting the various regulatory activities. Both annual administrative penalties and notifications of accumulating points on local health care facilities’ violations decreased considerably, with complaints against health care facilities reducing. As our study identified significant challenges, including regulatory fragmentation and no risk-based approach used, we offer recommendations to develop new policies and establish new mechanisms for better regulation.

## Introduction

Government regulation, acting as a fundamental form of social governance, has been used to ensure the quality, safety and equity of health care worldwide [1–4]. While non-governmental organizations play a predominant role in regulating health care industries within some nations, the governmental regulatory system radically prevails in many countries, which seems to be a direct reflection of differences in the political, economic, social and cultural context [5, 6]. However, in the health care arena, as in other sectors, government regulation is indispensable, as the unique characteristics of health care as both a social and private good reinforces the importance of active government regulation [7].

In mainland China, the governmental regulatory system has a pivotal role in improving legal compliance, ensuring patient safety and advancing health care quality across a variety of providers. Historically, subsequent to the founding of the People’s Republic of China, the first national regulatory scheme of health care organizations, *the Interim Regulation on the Administration of Hospitals and Clinics,* was enacted by the Government Administration Council of the Central Government (now the State Council) in 1951; this scheme was introduced to impose a set of mandates on licensing private hospitals and clinics, overseeing clinical practice, exercising enforcement over violators and empowering health departments of governments at the central, provincial, municipal and county levels to regulate health care entities providing medical services [8]. The enactment of *the Regulation on the Administration of Medical Institutions (RAMI)* issued by the State Council in 1994, which has been acting as the backbone of the legislation for institutional regulation to the present, strengthened external oversight of health care organizations and reinforced the accountability of governmental overseers [9]. The governmental regulatory system was further intensified by the issuance of *the Rules for the Development of Health Supervision Systems* in 2005, *the Opinions for the Enhancement of Health Supervision on a Comprehensive Basis* in 2013 and *the Opinions for Further Strengthening the Comprehensive Administrative Supervision and Enforcement in the Health Sector* in 2015 by the National Health Authority [10–12]. In 2016, as one of the five national fundamental health care institutions, the policy of *Promotion of the Development of a Comprehensive Regulatory System* was proposed by the National Health Summit attended by top leaders of China [13]. To this end, *Opinions for the Reform and Development of Comprehensive Supervision Regime in the Health Care Industry* was released by the General Office of the State Council in 2018 to establish more rigorous and effective government regulation within the health care sector [14]. However, few empirical studies have investigated the status of Chinese governmental oversight of health care facilities at either the national or provincial level in terms of regulatory arrangements and approaches.

Shanghai, the most populous city in China, has a population of 24.2 million with a life expectancy of 83.2 for registered residents by the end of 2016 [15]. Due to the massive population and aging society, the city features abundant health care resources. As demonstrated by official statistics on these regulated organizations (Table 1), the local medical system is increasingly complex, with a growing number of health care providers in recent years. However, the prevalence of poor compliance, deficiencies and even illegal conduct among some care organizations, especially private facilities, urged the local government to reinforce its administrative regulatory strength [16–18]. For instance, two local private hospitals that received negative media exposure in 2018 were confirmed to have many violations after official investigations, such as employing unqualified medical staff to perform clinical duties, committing out-of-scope practice and contravening provisions of the Administration of Radiological Diagnosis and Treatment [19, 20].

**Table 1:** Statistics on health care facilities in Shanghai between 2013 and 2017

Form of facilities	Number of health care facilities				
	2013	2014	2015	2016	2017
Hospital	308	310	313	321	326
Nursing home	20	22	25	28	37
CHC	1009	1028	1035	1039	1009
Village clinic	1342	1310	1271	1218	1187
Outpatient department	574	610	633	683	831
Clinic	1503	1530	1518	1482	1439
Nursing station	11	14	23	48	108
Freestanding clinical lab	6	6	7	11	24
Miscellaneous facility	54	55	57	55	55
Total	4827	4885	4882	4885	5016

Source: Shanghai Statistical Yearbook 2014–2018

Health care facilities refer to the medical institutions that deliver diagnostic and/or therapeutic procedures for patients and other care receivers, excluding some types of public health settings providing nonclinical services, such as the Centers for Disease Control and Prevention, Health Education Institutions, Blood Centers and Stations, etc. CHC refers to Community Health Care Centers and Stations. Clinic includes outpatient clinics, health posts and infirmaries. Miscellaneous facility refers to specialized disease prevention and treatment institutions, maternity and child healthcare institutions, sanatoriums, medical emergency centers and first-aid stations

Over several decades, the oversight of health care facilities in Shanghai had, for the most part, been carried out directly by the municipal and district government departments. Non-governmental oversight has been playing an auxiliary role in supervising local medical institutions in that the professional organizations, e.g., Shanghai Hospital Association and Shanghai Association for Non-Government Medical Institutions, have no regulatory powers without legal authorization. *The Administrative Measures for the Administration of Medical Institutions in the Shanghai Municipality (AMAMI)* promulgated by the municipal government in 1997 acts as an important local regulatory scheme [21]. According to AMAMI, the Municipal Supervisory Office of Medical Institution (MSOMI) was established within the Shanghai Municipal Health Bureau (now Shanghai Municipal Health Commission) to serve as a designated and dependent inspectorate to perform inspections and investigations in medical facilities [22]. In 2001, MSOMI’s regulatory functions were incorporated into the Shanghai Municipal Health Supervision Agency (now the Agency for Inspection and Supervision, Shanghai Municipal Health Commission) to exert more vigorous oversight on medical organizations [23]. Additionally, all district Health Supervision Agencies (HSAs) were delegated a remit successively to oversee local medical practice afterwards. Thus, as independent public institutions rather than governmental departments, HSAs became the main actors of government regulation over health care facilities.

Unlike some Western countries, regulatory arrangements for health care organizations in mainland China vary slightly from province to province. Thus, it would be of great value to understand the Chinese governmental regulatory landscape of health care, which is now the increasing focus of national legislation and reform, by examining the distinct approaches to regulation in Shanghai, a provincial-level city. Here, we explored the regime and main activities of government regulation over health care facilities in Shanghai, compared it with the governmental stewardship in Hong Kong and Taipei, two major Chinese cities under different social and political regimes than Shanghai, and identified challenges and offered recommendations for better regulation.

## Methods

To explore the regulatory regime and main activities of government regulation of medical institutions in Shanghai, we reviewed the literature related to regulations prevalent in the city and assessed the local data on regulatory activities and complaints against health care facilities between 2013 and 2017. We also compared the main characteristics in relation to government regulation on health care facilities between Shanghai, Hong Kong and Taipei through a literature review to improve understanding of Shanghai’s government regulation system.

### Literature search strategy

#### Government regulation of health care facilities in Shanghai

A literature search was conducted using the following databases: China National Knowledge Infrastructure (CNKI), Wanfang Data, Chinese Bio-Medicine database (CBM), Pubmed and Web of Science. We retrieved and reviewed legal norms and policy documents (Table 2) from official websites of related organizations and government bodies, such as the State Council, the National Health Commission (formerly Ministry of Health), the Shanghai Municipal Government and the Shanghai Municipal Health Commission (formerly Shanghai Municipal Health Bureau).

**Table 2:** Review of legal norms and policy documents on government regulation of health care facilities in Shanghai

No.	Name of the document	Issued by	Issued Year	Legal hierarchy
1	Regulation on the Administration of Medical Institutions	The State Council	1994	Administrative regulation
2	Rules for the Development of Health Supervision Systems	Ministry of Health	2005	Departmental rule
3	Opinions for the Enhancement of Health Supervision on a Comprehensive Basis	National Health Commission	2013	National normative document
4	Administrative Measures for the Administration of Medical Institutions in the Shanghai Municipality	Shanghai Municipal People’s Government	1997	Rule of local government
5	Notice on Issuing the Interim Measures for the Administration of Cumulative Points regarding Medical Institutions’ Illegal Conduct in Shanghai	Shanghai Municipal Health Bureau	2006	Local departmental document
6	Opinions on Further Development of the Health Supervision System in Shanghai	Shanghai Municipal Health Bureau	2007	Local departmental document
7	Measures for the Administration of Cumulative Points regarding Medical Institutions’ Illegal Conduct in Shanghai	Shanghai Municipal Health Bureau	2012	Local departmental document

#### Government regulation of health care facilities in Hong Kong and Taipei

In addition to employing literature databases, including CNKI, Wanfang Data, CBM, Pubmed and Web of Science, we retrieved and reviewed related public information, legal and policy documents and reports (Table 3) from official websites of the Hong Kong Department of Justice, the Hong Kong Hospital Authority, the Hong Kong Department of Health as well as the Legislative body in Taiwan Province and the Department of Health of Taipei City Government.

**Table 3:** Review of legal and policy documents and reports on government regulation of health care facilities in Hong Kong and Taipei

No.	Name of the document	Issued by	Issued Year
1	Hospital Authority Ordinance	Hong Kong Legislative Council	1990
2	Hospital Authority Annual Report 2017–2018	Hong Kong Hospital Authority	2018
3	Private Healthcare Facilities Ordinance	Hong Kong Legislative Council	2018
4	Department of Health Annual Report 2014/15	Hong Kong Department of Health	2015
5	Medical Care Act	Legislative body in Taiwan Province	1986
6	Public Health of Taipei City 2017 Annual Report	Department of Health of Taipei City Government	2018

### Data sources

Data were collected from two sources. The number of health care facilities in Shanghai was collected from the Shanghai Statistical Yearbook 2014–2018. Data about on-site inspections, administrative penalties, cumulative points imposed on health care facilities and complaints against health care facilities were derived from the centralized data bank established by the Agency for Inspection and Supervision, Shanghai Municipal Health Commission (AIS, HSA at the municipal level). The database encompasses all data regarding inspections conducted, punitive activities implemented and complaints received by HSAs at both the municipal and district levels.

### Statistical analysis

We analyzed annual regulatory activities of HSAs and complaints against health care facilities in Shanghai during the study period using descriptive statistical analysis. All statistical analyses were conducted with Stata 14.1 for Windows.

## Results

### The bureaucratic structure of the government regulation of health care facilities in Shanghai

**Fig. 1:**
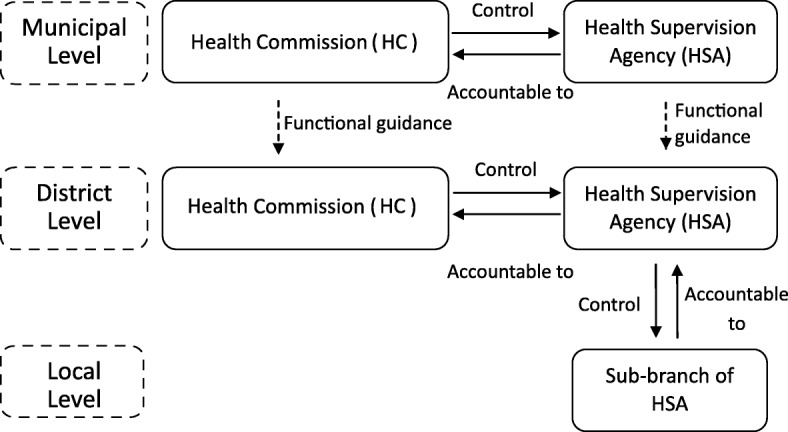
The bureaucratic structure of the government regulation of health care facilities in Shanghai. SOURCE: *Opinions on Further Development of Health Supervision System in Shanghai* issued by the Shanghai Municipal Health Bureau in 2007 (http://wsjkw.sh.gov.cn/zhjd/20180526/38386.html). Sub-branch of HSA refers to a substation or squadron established by a HSA at the district level that acts as a sub-inspectorate at the township level and monitors local health care organizations and reports to the HSA in some suburban districts

The administrative oversight of health care facilities, acting as a basic, fundamental and essential form of regulation, aims to ensure patient safety and advance care quality by evaluating the compliance of regulated entities with related laws, regulations, rules and provisions formulated by various legislators and by imposing punitive actions on offenders. The local health authorities (now the Health Commissions, HCs) at the municipal and district levels, legally speaking, have been serving as regulatory bodies over a wide range of different types of health care organizations. However, HCs normally do not themselves inspect health care facilities to check their legal compliance currently; instead, they delegated this task to HSAs at municipal and district levels, respectively [10, 24]. As both a subordinate agency of the HC and an independent public institution without enforcement powers, HSA acts as a regulatory executive in performing inspections, investigating complaints, identifying deficiencies in regulated facilities and initializing and implementing punitive processes on behalf of the HC; the HC controls and oversees the regulatory activities executed by the HSA to make sure that supervision has been implemented appropriately [24]. As illustrated in Fig. 1, the current governmental regulatory system is a combination of municipal and district governmental regulators.

### Main activities of government regulation over health care facilities in Shanghai

#### On-site inspection: the primary regulatory activity

To determine whether health care organizations conform to legal requirements, HSAs rely primarily on on-site inspections [25, 26]. Although other information sources (such as suspected illegal conduct reported by other governmental departments or public complaints) has been used, site visits are the primary regulatory activities used to monitor and check institutional compliance with standards and regulations [18]. These supervisory activities are generally unannounced and are conducted by an inspector team of at least two staff from the HSA [27]. As a relatively deterrence-oriented approach, regulatory inspection can start at any time and focus on identifying and categorizing deficiencies or illegal provider conduct. However, the duration for an on-site inspection in a regulated organization lasts usually no more than 1 day with the cooperation of HSA supervisors, and the inspectors spend their time visiting clinical departments and services, reviewing records of care and related documents and reports, and occasionally interviewing the staff and patients [28].

Regarding the scope of regulatory inspection, HSAs have a broad range of oversight over the routine practices of health care facilities pursuant to the legislation [10, 11], including the following: 1) the functional and geographical scope of the practice [9, 21]; 2) the qualifications of the various professional staff [9, 21]; 3) the clinical use of drugs and prescription [29]; 4) the clinical use of blood [30]; 5) the purchase and clinical use of large-scale medical equipment [31]; 6) the clinical use of medical techniques [32]; 7) the testing quality of clinical labs [33]; 8) the procedures and environment of radiological diagnosis and treatment [34]; 9) the prevention and control of infectious diseases along with disposal of medical waste [35] and 10) the medical advertisements released by the regulated facilities [36]. These compliance statuses within health care facilities are normally inspected by different inner divisions of HSA separately and respectively rather than on a comprehensive basis.

As presented in Table 4, the overall annual count of inspections conducted by HSAs of all health care facilities in Shanghai decreased from 18,128 to 13,351 during the study period, with the total regulated facilities increasing from 4827 to 5016. As a result, the average annual number of governmental inspections that an individual care facility underwent decreased by an average of 1.1, from 3.8 to 2.7 average inspections per year. Some facilities underwent more than forty administrative inspections in a calendar year.

**Table 4:** Statistics of on-site inspections conducted by HSAs in Shanghai between 2013 and 2017

Year	Number of facilities^a^	On-site inspections of health care facilities per year		
		Total	Mean	Max.
2013	4827	18128	3.8	67
2014	4885	17075	3.5	68
2015	4882	14934	3.1	41
2016	4885	12953	2.7	42
2017	5016	13351	2.7	45

SOURCE: Authors’ analysis of data from the On-site Inspection Database established by the Agency for Inspection and Supervision, Shanghai Municipal Health Commission

^a^Shanghai Statistical Yearbook 2014–2018

#### Administrative penalties: the main punitive enforcement approach

When illegal conduct is identified in a regulated facility, the HSA will generally impose administrative penalties on behalf of the HC to ensure that the regulated facility addresses the problems, regains compliance with regulations and can avoid the same lapses in the future [9, 10, 26]. A broad range of administrative punitive actions has been introduced according to the penalty provisions of the laws, regulations and rules, including disciplinary warnings, punitive fines, confiscations of illegal gains, confiscations of drugs and medical devices used for the provision of illegal care, suspensions of the clinical practice and even revocations of the license for practice [9, 21].

The annual overall administrative penalties imposed by HSAs on health care facilities in Shanghai decreased by 24.8% from 1265 to 951 during the study period, and the number of revocation cases increased (Table 5).

**Table 5:** Administrative penalties and punitive actions imposed by HSAs on health care facilities in Shanghai between 2013 and 2017

Year	Number of Penalties	Type of punitive actions				
		Warning	Fine	Confiscation	Suspension	Revocation
2013	1265	747	816	26	0	3
2014	1203	745	752	18	0	3
2015	1065	597	690	16	0	2
2016	960	544	672	29	1	7
2017	951	528	664	24	0	10

SOURCE: Authors’ analysis of data from the Administrative Penalty Database established by the Agency for Inspection and Supervision, Shanghai Municipal Health Commission;

One penalty can contain one or more punitive actions, such as a disciplinary warning and punitive fine, imposed on a health care facility that engaged in illegal actions. Warning refers to the number of disciplinary warnings. Fine refers to the number of punitive fines. Confiscation refers to the number of confiscations of illegal gains and drugs and medical devices used for the provision of illegal care. Suspension refers to the number of suspensions of clinical practice of health care facilities; Revocation refers to the number of revocations of the health care facilities’ license for entire or partial clinical practice

#### Cumulative points system: a complementary punitive approach

In addition to administrative penalties, a cumulative points system was first adopted in 2007 in Shanghai as a complementary punitive approach for medical facilities committing infractions [37, 38]. When an individual facility is incompliant with *the Administrative Measures for the Clinical Use of Antibacterial Drugs*, for example, the responsible health authority will accumulate two points against the provider’s license by issuing a formal administrative notification in addition to imposing administrative sanctions. There are many violations that can result in a facility having points applied to its license according to the rules of this cumulative points system [38–40]. Once a facility with over 100 beds has or exceeds 36 points accumulated against its license in a 36-month period of time or once a smaller facility has 12 points accumulated within 12 months, that facility is subject to a period of suspension for 1 to 6 months [40].

During the study period, the number of annual overall notifications for accumulating points issued by HSAs decreased by 40.7%, from 801 to 475, and the total points accumulated against providers’ licenses and the number of health care facilities with points and in suspension also decreased, as shown in Table 6.

**Table 6:** Cumulative points imposed by HSAs on health care facilities in Shanghai between 2013 and 2017

Year	Number of Notifications	Total points accumulated	Health care facilities having points	Health care facilities in suspension
2013	801	1952	479	15
2014	893	1960	525	15
2015	683	1419	448	3
2016	635	1328	416	5
2017	475	1062	306	2

SOURCE: Authors’ analysis of data from the Administrative Cumulative Points Database established by the Agency for Inspection and Supervision, Shanghai Municipal Health Commission;

Notifications refer to the number of notifications issued by HSAs for accumulating points against providers’ licenses. Total points accumulated refer to aggregate points accumulated against providers’ licenses by HSAs in a year. Health care facilities having points refer to the number of facilities having points applied to their licenses in a year. Health care facilities in suspension refer to the number of facilities subject to a period of suspension for one to six months due to cumulative points

#### Complaints investigations: an important fact-finding mechanism

HSAs also have an important duty to investigate complaints with regard to the practices of health care facilities [41, 42]. Through a unified hotline and online platform within the Health Supervision Authority, the regulatory agencies received and handled a variety of complaints filed by patients, local residents and social organizations, which have commonly served as an important fact-finding mechanism for probes into illegal conduct [43].

As shown in Table 7, the total annual complaints received by HSAs in Shanghai decreased by 29.1%, from 763 to 541, within the study timeline. The most frequent complaints against health care facilities received by HSAs between 2013 and 2017 were unqualified medical staff, illegal advertisement and fraud by hiring decoys.

**Table 7:** Complaints against health care facilities received by HSAs in Shanghai between 2013 and 2017

Year	Complaints received	Top three kinds of complaints received and their ranks in a year		
		1	2	3
2013	763	Fraud by hiring decoys	Illegal advertisement	Unqualified medical staff
2014	589	Illegal advertisement	Unqualified medical staff	Fraud by hiring decoys
2015	501	Unqualified medical staff	Illegal advertisement	Out-of-scope practice
2016	592	Unqualified medical staff	Illegal advertisement	Fraud by hiring decoys
2017	541	Unqualified medical staff	Fraud by hiring decoys	Illegal advertisement

SOURCE: Authors’ analysis of data from the Complaints Registration Database estalished by the Agency for Inspection and Supervision, Shanghai Municipal Health Commission

Complaints received refer to the number of complaints received by HSAs in a year. Fraud by hiring decoys refers to an illegal activity in which a health care facility cheats patients by hiring decoy employees to convince patients to receive unnecessary services (in Chinese: *Yituo*). Unqualified medical staff refers to a health care facility employing unqualified medical staff to provide medical services. Illegal advertisement refers to a health care facility releasing unapproved or mendacious medical advertisements via social media

### Comparative analysis of 3 Chinese cities in their government regulation of health care facilities

#### Hong Kong special administrative region, China

Under the *Hospital Authority Ordinance*, the Hospital Authority (HA), a statutory non-governmental body corporate established in 1990, is responsible for managing and controlling all public hospitals in Hong Kong [44–46]. The HA provides systematic coverage of the internal control and risk management systems to oversee the operational and financial performance of public healthcare facilities and enhances governance over them through 32 Hospital Governing Committees and 11 functional committees [46, 47]. In accordance with *the Private Healthcare Facilities Ordinance*, the Hong Kong Department of Health was authorized to supervise private hospitals, day procedure centers, clinics and health services establishments [48]. The compliance of these private institutions is monitored through field inspections, scrutiny of institutional activities and complaint statistics, investigation of medical incidents and handling of complaints [49]. As specified by the Department, the on-site inspections of private hospitals must occur at least twice a year per institution and at least once a year for each nursing home [50]. In 2014, 244 inspections to a total of 76 private facilities were conducted by the Department [49]. If a private facility is not in compliance with the ordinance, its license for practice may be suspended or cancelled; if the licensee or chief medical executive of a facility has been convicted of a criminal offense under this ordinance, he or she may be liable to a fine or imprisonment [48].

#### Taipei City, Taiwan Province, China

Unlike the regulatory regime in Hong Kong, the Taipei Municipal Department of Health was authorized the regulatory power to oversee all local public and private health care facilities under the Medical Care Act [51, 52]. According to this act, all medical care institutions shall accept inspections or data collection conducted by the responsible health authority regarding professional personnel, medical equipment, medical practices, sanitation and safety, and medical records. A facility that violates the legal provisions shall be subject to a warning, a fine, a suspension of practice or even a revocation of practice license [52]. It was reported by the Department of Health that a total of 781 medical violations were punished by the municipal health authority in 2017 [53].

As indicated in Table 8, we summarized the main characteristics of these three cities in relation to government regulation of health care facilities in terms of regulatory legislation, regulatory body, regulatory executive agency, regulatory hierarchy, significant regulatory activities and the person who performs routine regulatory activities. Hong Kong has a dual system of health care regulation for public and private health care facilities, while Shanghai and Taipei impose the same legal requirements on public and private organizations under a governmental regulatory regime. The governmental oversight of health care facilities in Shanghai is undertaken by the HSA as a regulatory executive agency, but the same tasks in Hong Kong and Taipei are conducted by the government departments or an authorized regulatory body. Among these three cities, on-site inspections are common regulatory activities for government regulation of health care facilities.

**Table 8:** A comparative analysis regarding government regulation of health care facilities in Shanghai, Hong Kong and Taipei

Characteristic	Shanghai	Hong Kong		Taipei
		Public healthcare facility	Private healthcare facility	
Main regulatory legislation	RAMI, AMAMI	Hospital Authority Ordinance	Private Healthcare Facilities Ordinance	Medical Care Act
Regulatory body authorized by legislation	Municipal and district HCs	Hospital Authority	Department of Health	Department of Health
Regulatory executive agency	HSAs at municipal and district level	No regulatory executive agency	No regulatory executive agency	No regulatory executive agency
Regulatory hierarchy	Municipal and district level	Municipal level	Municipal level	Municipal level
Some significant regulatory activities or measures	On-site inspections, Administrative penalties, Cumulative points, Complaints investigations	Internal control and risk management	Field inspections, Complaint investigations, Execution of suspension or cancellation of license	On-site inspections, Data collection, Administrative penalties
The person who performs regulatory activities	Specialized supervisors	Members of the functional committees	Civil servants	Civil servants

## Discussion

The government has a basic responsibility to ensure that providers are qualified and operate in the public interest [14, 54]. Regulation can be regarded as a significant approach to achieving this goal. Our study examined the structure and main activities of government regulation over health care facilities in Shanghai and compared it with the governmental regulatory systems in Hong Kong and Taipei. The results demonstrated trends in regulatory activities of government regulators and characteristics of these unique regulatory arrangements in Shanghai.

### Uniqueness of the government regulation of health care facilities in Shanghai

Compared with the regulatory regime in Hong Kong and Taipei, Shanghai established its unique regulatory executive agency, the HSA, which serves as a key actor within the governmental regulation system on behalf of the HC. To undertake its supervisory tasks, the regulatory agency has committed itself to developing highly specialized supervisors in place of ordinary civil servants and improving the ability to oversee medical practice. With the regulatory network at both municipal and district levels, the two-tiered regulatory hierarchy in Shanghai has been established to guarantee enhanced oversight of health care providers to safeguard patient safety and improve care quality compared to the one-tiered regulatory hierarchy in Hong Kong and Taipei.

### On-site inspections conducted by HSA in Shanghai

Inspections are widely used as means to monitor the health care institution’s compliance with the legal requirements. Although the results revealed that the annual overall inspections imposed by HSAs on health care facilities in Shanghai declined during the study period, the local governmental regulatory system still relies primarily on this regulatory activity as a significant surveillance measures to find deficiencies in the supervised organizations. The reason why annual overall count of inspections decreased may be related to the implementation of a random inspections policy at the national level since 2015, with the aim of streamlining administrative procedures and reducing unnecessary regulatory burden [55, 56]. This policy encouraged the governmental overseers to conduct ad hoc visits on a random sample of the regulated facilities instead of delivering an arduous oversight on a universal basis.

While annual overall counts of inspections against health care facilities in Shanghai decreased over the study period, it is still worth noting that some local medical institutions were inspected much more frequently. As demonstrated in Table 4, some facilities were inspected over forty times yearly. We believe there may be the following reasons accounting for the situation. First, these multiple inspections would be a consequence of the combination of oversight at the municipal and district levels, which forced the regulated entities to face overlapping inspections and field scrutiny. Second, on-site inspections at health care facilities are normally undertaken separately by various inspectorates from different inner divisions of an HSA, which could also contribute to an increased and onerous regulatory burden on local health care organizations. In addition, some facilities underwent over forty inspections annually presumably due to the wider breadth of their clinical activity that elicited more supervisory visits from different divisions of HSAs. Thus, we argue that there existed fragmentation across the local governmental regulatory arrangements, which may result in higher regulatory costs, some conflict or confusion between different regulators and weakening regulatory oversight, as observed in the literature [1, 57].

In addition to the regulatory fragmentation, we remain unsure whether these supervised organizations deserved such frequent administrative supervision because there is no risk-based assessment approach introduced by local governmental regulators. As reported by prior studies, risk assessment is an essential measure of directing regulatory resources, through which the government can end unnecessary inspections on less risky businesses and identify businesses that need more inspections [58–60]. The Ministry of Health in Singapore introduced a Risk-Based Licensing Framework (RBLF) in 2013 for all medical and dental clinics so as to reduce unnecessary regulatory burden; under an updated version of RBLF effective in 2018, the Singaporean governmental regulator will no longer conduct inspections before license renewals for the clinics with good compliance [61].

### Punitive activities imposed and complaints received by HSAs in Shanghai

We found that the annual overall administrative penalties and cumulative points imposed by HSAs on health care facilities in Shanghai decreased considerably during the study period, with fewer complaints against health care facilities received by HSAs. As shown in Table 7, the most frequent complaints were unqualified medical staff, illegal advertisement and fraud by hiring decoys, which closely related to the occurrences of illegal behaviors committed by medical institutions. Therefore, although the decline of administrative penalties and cumulative points might be associated with the reduced number of inspections of HSAs, we argued that, given fewer complaints received, the decreased administrative sanctions very likely reflected a better legal compliance among local health care facilities.

### Strengths and limitations of the study

Our study employed administrative panel data from 2013 to 2017 to explore the status and main activities of overseeing health care facilities in Shanghai based on a literature review, compared the governmental regulatory systems in Hong Kong and Taipei to provide policy implications for better regulation in Shanghai and also offer valuable reference for elsewhere in China and other developing countries. As far as we know, this study is the first to examine the regulatory regime and detailed approaches to governmental regulation over health care facilities in China.

However, the current study also has limitations. First, because of incomplete data, data about on-site inspections, administrative penalties, cumulative points imposed on health care facilities and complaints against health care facilities in Shanghai were only accessible from 2013 onward. However, we also arrived at several useful policy implications by analyzing data available. Second, we did not examine characteristics of the health care facilities undergoing frequent inspections and administrative sanctions due to a lack of details about these entities in the regulatory data bank. We will try to complement related information in future research by redesigning the extended fields in the centralized data bank of AIS. Third, our study did not examine regulatory functions and activities of other governmental regulators in Shanghai, e.g., Health Insurance Bureau, Food and Drug Administration, as this study focused on regulatory structure and approaches within the health sector. We plan to conduct a cross-sector analysis with regard to government regulation of health care facilities in the following research.

## Conclusions

Shanghai has established its unique governmental regulatory system which had played a crucial role in regulating the health care facilities. In regard to the regulatory activities, we found that both annual administrative penalties and notifications of accumulating points on local health care facilities’ violations from 2013 to 2017 decreased considerably, with complaints against health care facilities reducing by 29.1%. These data of regulatory activities indicated that local health care facilities were probably in better compliance with the legal requirements. However, the regulatory system still faces some significant challenges, such as regulatory fragmentation and absence of a risk-based regulatory approach, although overall inspections conducted by HSAs decreased during the study period. These identified problems may be not unique to the health care governance in Shanghai, and it seems that much could be learned by elsewhere in China and other developing countries.

Our findings have significant policy implications and serve to provide insights into the following recommendations. A reform of the regulatory system will, for one thing, be needed to reshape the structures and functions of the governmental regulatory agencies to address the fragmentation by reducing local regulatory hierarchies and exerting inspections on a comprehensive basis. For another, regulation should be responsive and precise. More inspections should be targeted at those providers whose compliance is poor based on a risk-based regulatory approach. An important next step is to develop new policies to transform the regulatory regime and establish new mechanisms for better regulation. Future studies should foremost focus on how to establish a reasonable risk-based methodology to implement differentiated oversight of local health care facilities.

## Abbreviations

AIS: Agency for Inspection and Supervision, Shanghai Municipal Health Commission
AMAMI: Administrative Measures for the Administration of Medical Institutions in the Shanghai Municipality
CBM: Chinese Bio-Medicine database
CNKI: China National Knowledge Infrastructure
HA: Hospital Authority Ordinance of Hong Kong
HC: Health Commission
HSA: Health Supervision Agency
MSOMI: Municipal Supervisory Office of Medical Institution
RAMI: Regulation on the Administration of Medical Institutions
RBLF: Risk-Based Licensing Framework
